# Regulation of Expression of the *TIR-Containing Protein C* Gene of the Uropathogenic *Escherichia coli* Strain CFT073

**DOI:** 10.3390/pathogens10050549

**Published:** 2021-05-01

**Authors:** Julia Ittensohn, Jacqueline Hemberger, Hannah Griffiths, Maren Keller, Simone Albrecht, Thomas Miethke

**Affiliations:** 1 Medical Faculty of Mannheim, Institute of Medical Microbiology and Hygiene, University of Heidelberg, Theodor-Kutzer-Ufer 1-3, 68167 Mannheim, Germany; julia.ittensohn@umm.de (J.I.); Jacqueline.Hemberger@medma.uni-heidelberg.de (J.H.); Hannah.Griffiths@medma.uni-heidelberg.de (H.G.); Maren.Keller@medma.uni-heidelberg.de (M.K.); Simone.Albrecht@medma.uni-heidelberg.de (S.A.); 2 Medical Faculty of Mannheim, Mannheim Institute for Innate Immunoscience (MI3), University of Heidelberg, Ludolf-Krehl-Str. 13-17, 68167 Mannheim, Germany

**Keywords:** uropathogenic *Escherichia coli*, virulence, promoter region, hydrogen-Ion concentration, glucose

## Abstract

The uropathogenic *Escherichia coli* strain CFT073 causes kidney abscesses in mice Toll/interleukin-1 receptor domain-containing protein C (TcpC) dependently and the corresponding gene is present in around 40% of *E. coli* isolates of pyelonephritis patients. It impairs the Toll-like receptor (TLR) signaling chain and the NACHT leucin-rich repeat PYD protein 3 inflammasome (NLRP3) by binding to TLR4 and myeloid differentiation factor 88 as well as to NLRP3 and caspase-1, respectively. Overexpression of the *tcpC* gene stopped replication of CFT073. Overexpression of several *tcpC*-truncation constructs revealed a transmembrane region, while its TIR domain induced filamentous bacteria. Based on these observations, we hypothesized that *tcpC* expression is presumably tightly controlled. We tested two putative promoters designated P1 and P2 located at 5′ of the gene c2397 and 5′ of the *tcpC* gene (c2398), respectively, which may form an operon. High pH and increasing glucose concentrations stimulated a P2 reporter construct that was considerably stronger than a P1 reporter construct, while increasing FeSO_4_ concentrations suppressed their activity. Human urine activated P2, demonstrating that *tcpC* might be induced in the urinary tract of infected patients. We conclude that P2, consisting of a 240 bp region 5′ of the *tcpC* gene, represents the major regulator of *tcpC* expression.

## 1. Introduction

Urinary tract infections are frequent, often recurrent and most often caused by uropathogenic *Escherichia coli* (UPEC). Expanding antibiotic resistance hampers successful treatment of urinary tract infections with UPECs. Therefore, exploration of the host-pathogen interaction is crucial for the elucidation of new treatment mechanisms and approaches. We detected a new class of virulence factors in the uropathogenic *E. coli* strain CFT073 and in *Brucella* spp., which impair Toll-like receptor (TLR) signaling [1]. By binding to myeloid differentiation factor 88 (MyD88) and TLR4 they impair the secretion of pro-inflammatory cytokines such as TNF-α and IL-6 [1,2]. Other groups detected structurally similar virulence factors with almost identical mechanisms in human pathogens such as *Salmonella enterica* subsp. *enterica* Serovars Enteritidis, Dublin, Gallinarum, *Yersinia* spp., *Brucella* spp., *Staphylococous aureus* MSSA476, *Enterococcus faecalis*, but also in non-pathogens such as *Paracoccus denitrificans* [3,4,5,6,7,8,9,10,11]. At least some of these TLR-inhibiting virulence factors significantly increase disease severity in different murine infection models [1,3,4,6,12].

We also described that the Toll/interleukin-1-receptor domain (TIR)-containing protein C (TcpC) from CFT073 impairs the NACHT leucin-rich repeat PYD protein 3 (NLRP3) inflammasome by binding to NLRP3 and caspase-1 [13]. This event prevents the prion-like condensation of apoptosis-associated speck-like protein (ASC), but also of caspase-1 to form intracellular specks, the hallmark of NLRP3 inflammasome activation. Consequently, the maturation and secretion of IL-1β is prevented [13].

Recently, TcpC from *E. coli* Nissle was reported to strengthen epithelial barrier function by stimulating PKCζ and ERK1/2 signaling in HT-29/B6 cells demonstrating another influence of TcpC on host cells [14]. Moreover, outer membrane vesicles and soluble factors, such as TcpC, released by *E. coli* Nissle as well as ECOR63 enhanced barrier function in intestinal epithelial cells [15].

The *tcpC* gene is only found in the phylogenetic group B2 of extra-intestinal *E. coli* strains (ExPECs) and is chromosomally located in the *serU* island, which co-segregates with the high pathogenicity island (HPI) of ExPECs [16]. The latter finding indicated that the *serU*-island and the HPI were transferred *en bloc* between ExPECs [16]. Around 40% of UPECs isolated from patients suffering from pyelonephritis carry the *tcpC* gene, while this gene is only present in 21% of UPECs responsible for cystitis, in 16% during asymptomatic bacteriuria and in only 8% of commensal *E. coli* strains in stool, respectively [1]. Thus, the frequency of *tcpC*^+^ UPECs correlates with disease severity in humans [1,16].

Structurally, TcpC consists of a Toll/Interleukin-1 receptor (TIR) domain, which is located in the C-terminal half of the molecule (bp 508–924) and interacts with the TLR-signaling cascade and the NLRP3 inflammasome [1,13]. We crystallized the homologous domain of TcpB from *Brucella* spp., which shares a 55% amino acid sequence identity to the TIR domain of TcpC, and demonstrated that the tertiary structure of the bacterial TIR domain has an arrangement of five central β-sheets and five surrounding α-helices which characterizes eukaryotic TIR domains [17]. TcpC but not TcpB is predicted to also contain a N-terminal transmembrane domain [1].

Although we published earlier that co-culture of RAW264.7 cells with CFT073 induced the production of TcpC, its mechanism of induction is still unknown and a detailed analysis is missing [1]. This is an important issue, since on the one hand the secreted TcpC could act in infected humans as an immunosuppressant during urosepsis with UPECs such as CFT073. Indeed, systemic application of TcpC was used to successfully treat mice suffering from autoimmune arthritis, which indicates the immunosuppressive potential of the molecule [18]. On the other hand, the potential consequences of TcpC expression on CFT073 itself are unknown as well. We thus determined the effect of TcpC overexpression on the multiplication of CFT073. Moreover, we explored the localization of different truncated constructs of TcpC in CFT073 and their influence on the morphology of the bacterium upon overexpression.

Based on the arrangement of genes surrounding the *tcpC* gene on the chromosome and their direction of gene transcription, the *tcpC* gene may form an operon with the 5′-located gene c2397 within the *serU* island, which encodes a hypothetical protein. Thus, promoter regions, which control transcription of *tcpC*, may be located at 5′ of the gene c2397 and/or c2398. We explored their functional relevance in the present study.

## 2. Results

### 2.1. Overexpression of TcpC Stops Growth and Provokes Filamentation of the Uropathogenic E. coli Strain CFT073

**Figure 1:**
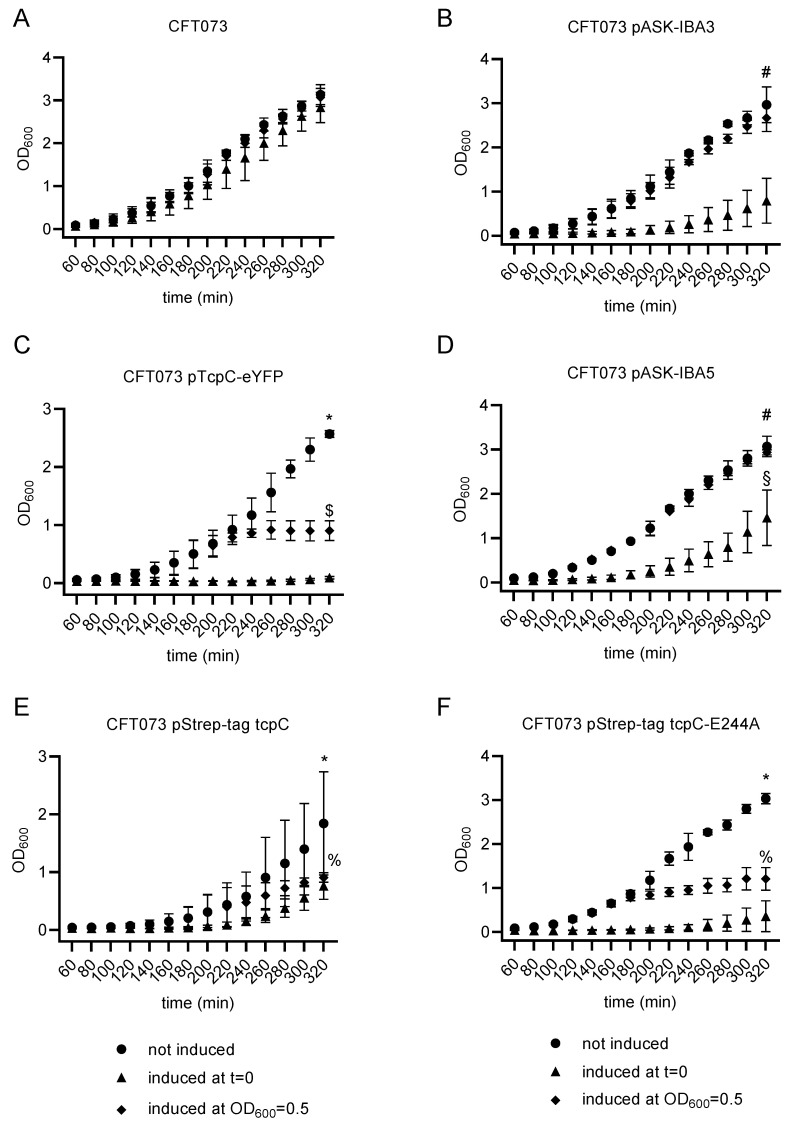
Overexpression of *tcpC* impairs growth of the uropathogenic *E. coli* strain CFT073. We used untransformed CFT073 as ATc control (**A**), CFT073 transformed with pASK-IBA3plus (**B**) as empty vector control or with *tcpC* fused to *eYFP* (**C**). We further transformed CFT073with pASK-IBA5plus (**D**) as empty vector control or with *tcpC* or E244A-mutated *tcpC* fused to a Strep-tag (**E**,**F**). The plasmids contained an ATc-inducible promoter. The plasmids were not induced (dots), induced with ATc (0.2 µg/mL) at the initiation of the culture (triangles) or induced at an OD_600_ of 0.5 (diamonds). Bacteria were cultivated in LB medium for the indicated periods. The graphs represent three independent experiments. * *p* < 0.05 not induced vs. induced at OD_600_ = 0.5 and not induced vs. induced at t = 0; ^#^ *p* < 0.05 not induced vs. induced at t = 0; ^§^ *p* < 0.05 CFT073 pASK-IBA5 induced at t = 0 vs. CFT073 pStrep-tag tcpC-E244A induced at t = 0; ^$^ *p* < 0.05 CFT073 pTcpC-eYFP induced at OD_600_ = 0.5 vs. CFT073 pASK-IBA3 induced at OD_600_ = 0.5; ^%^ *p* < 0.05 CFT073 pStrep-tag tcpC induced at OD_600_ = 0.5 vs. CFT073 pASK-IBA5 induced at OD_600_ = 0.5; CFT073 pStrep-tag tcpC-E244A induced at OD_600_ = 0.5 vs. CFT073 pASK-IBA5 induced at OD_600_ = 0.5; ANOVA post-hoc Tukey’s test.

To study the effects of TcpC expression on the UPEC strain CFT073, we transformed the bacterium with two different non-leaky plasmids, which allowed the anhydrotetracycline (ATc)-inducible expression of TcpC fused either to an enhanced yellow fluorescent protein (eYFP) or to a Strep-tag. ATc did not influence the growth of untransformed CFT073 significantly (Figure 1A). However, ATc induction of two empty vector controls demonstrated that the induction of the plasmids at the beginning of the culture period impaired the replication of CFT073 (Figure 1B,D). We did not observe growth inhibition when both plasmids were induced at an OD_600_ of 0.5 (Figure 1B,D). Growth of CFT073 was impaired when the *tcpC* gene was induced at the beginning of the culture and it stopped when the gene was induced after the culture reached an OD_600_ density of 0.5 (Figure 1C). In the latter case, growth of CFT073 significantly differed from the growth observed upon induction of the control plasmid pASK-IBA3 (Figure 1B,C). We were worried that the addition of eYFP might yield a toxic protein being responsible for the growth retardation (Figure 1C), however, the addition of the much smaller Strep-tag to TcpC again prevented growth of the bacterium, indicating that TcpC itself but not eYFP is responsible for growth retardation (Figure 1E). As discussed above, growth of CFT073 upon induction of the plasmid pStrep-tag tcpC or the corresponding control plasmid differed significantly if they were induced at an OD_600_ of 0.5 (Figure 1D,E). Since TcpC was reported to cause NAD^+^ loss in *E. coli*, which in turn impairs its growth, we generated an E244A-mutant of TcpC, which lacks the ability to cleave NAD^+^ [19]. However, E244A-mutated TcpC impaired the growth of CFT073 as efficiently as wild-type TcpC (Figure 1F).

**Figure 2:**
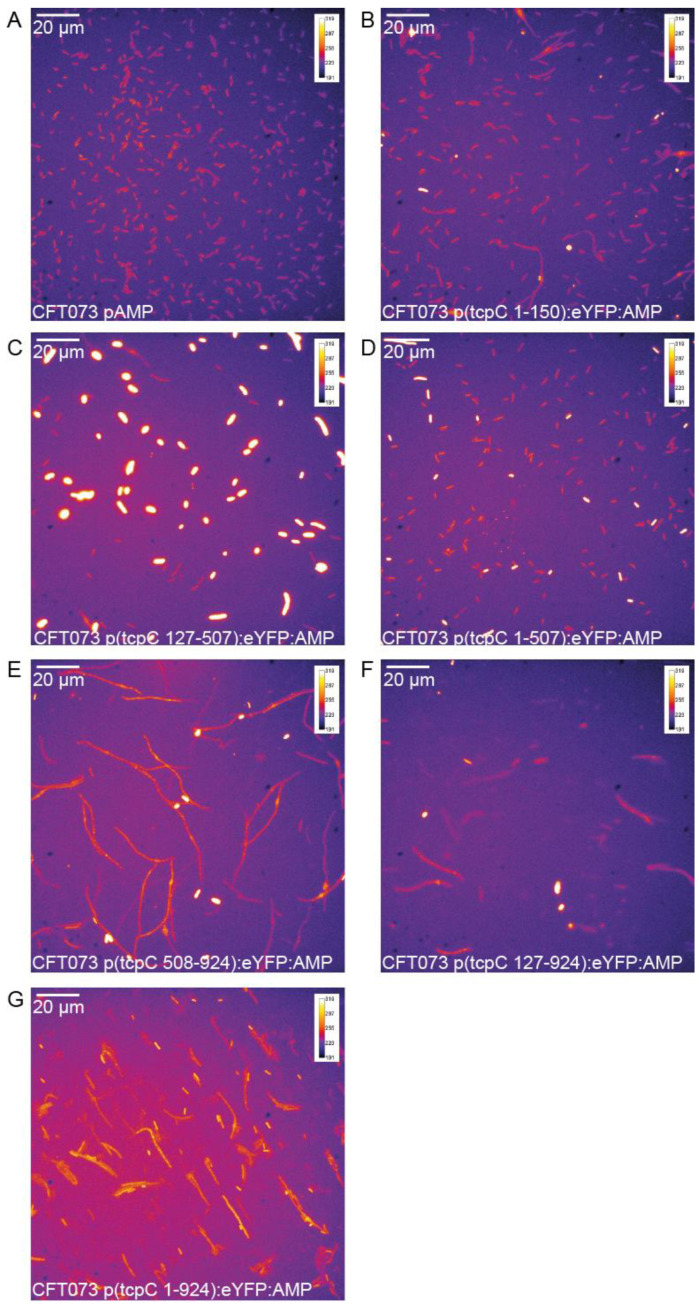
The TIR domain of TcpC induces filamented bacteria. CFT073 transformed with pAMP, which contained no eYFP, served as the negative control (**A**). We transformed CFT073 with the IPTG-inducible eYFP-reporter plasmids p(tcpC 1–150):eYFP:AMP (**B**), p(tcpC 127–507):eYFP:AMP (**C**), p(tcpC 1–507):eYFP:AMP (**D**), p(tcpC 508–924):eYFP:AMP (**E**), p(tcpC 127–924):eYFP:AMP (**F**) or p(tcpC 1–924):eYFP:AMP (**G**). We analyzed the expression of the different truncated or full-length constructs of *tcpC* as well as the morphology of the bacteria by fluorescence microscopy four hours post induction with IPTG. Bacteria were cultured in M9-minimal medium containing glucose, thiamine and nicotinic acid. We repeated the experiment once with identical results.

**Figure 3:**
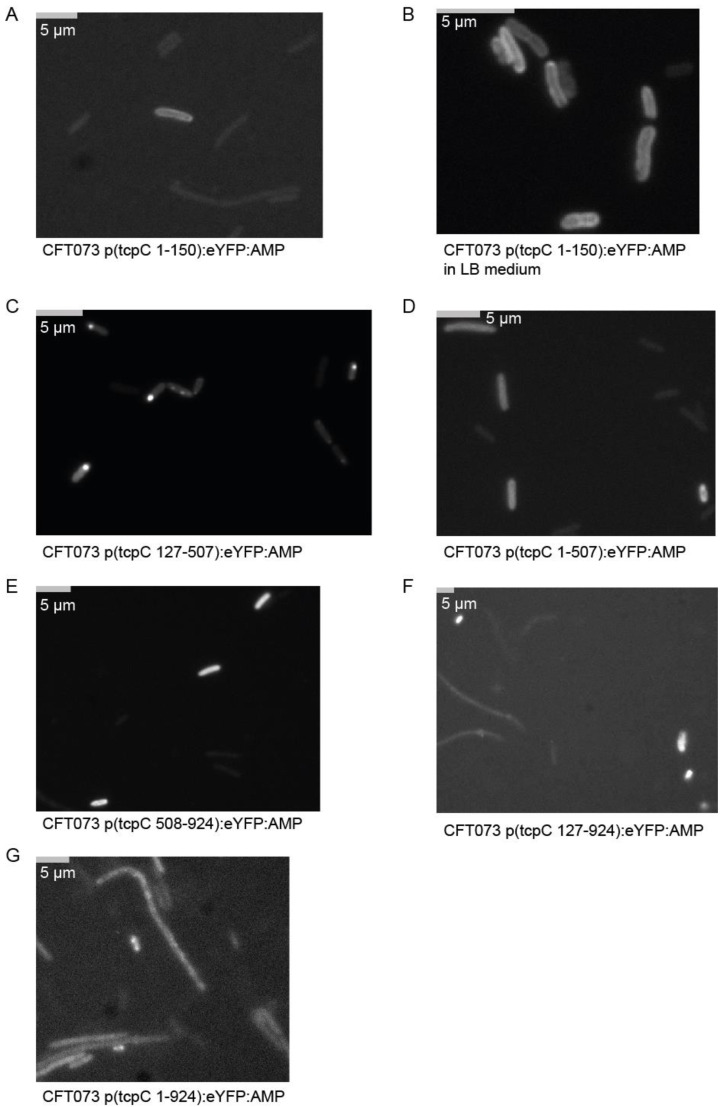
Intracellular distribution of TcpC four hours post induction. High-resolution (magnification ×1000) fluorescence-microscopy of the CFT073 transformants analyzed in Figure 2. Bacteria were transformed with the plasmids as indicated (**A**–**G**) and cultured in M9-minimal medium containing glucose, thiamine and nicotinic acid (**A**,**C**–**G**), except in (**B**) where LB medium was used. We repeated the experiment once with identical results.

**Figure 4:**
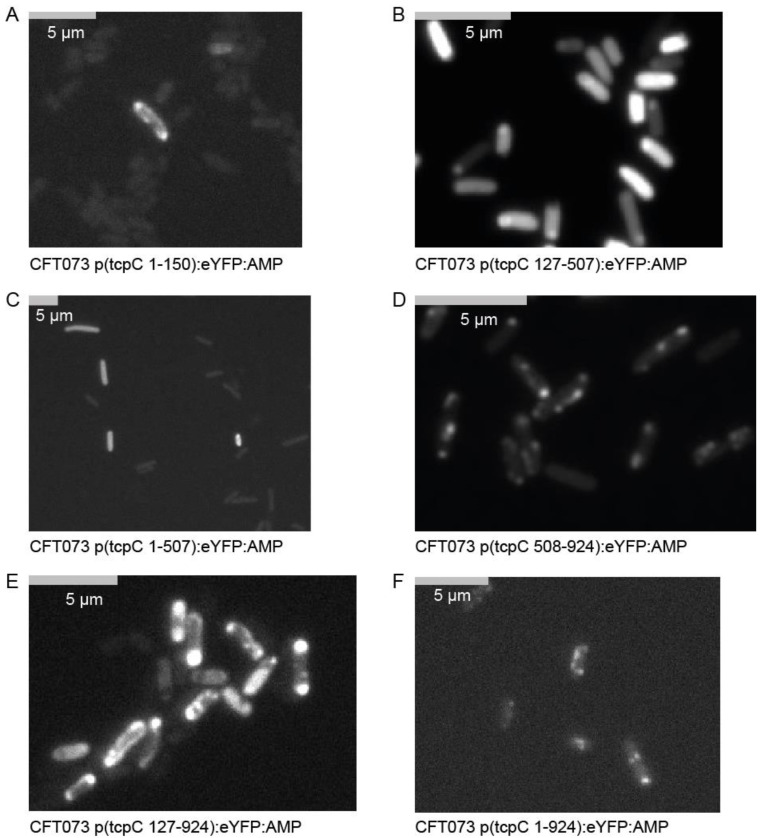
Intracellular distribution of TcpC 24 h post induction. High-resolution (magnification ×1000) fluorescence-microscopy of the CFT073 transformants analyzed in Figure S2. The plasmids used are indicated in (**A**–**F**). We repeated the experiment once with identical results.

We subsequently explored whether morphologic changes imposed on the bacterium accompany the negative influence of TcpC on bacterial growth and if so, which part of the molecule might be responsible for these changes. We constructed a series of truncated constructs of *tcpC* fused to *eYFP*. At four hours post induction of the different constructs major morphologic changes were imposed on CFT073. Thus, all plasmids containing the TIR domain (bp 508–924), i.e., p(tcpC 508–924), p(tcpC 127–924) and p(tcpC 1–924), caused strong filamentation of the bacteria (Figure 2, Supplementary Materials Figure S1). We observed this to a much lower degree or not at all with plasmids p(tcpC 1–150), p(tcpC 127–507) and p(tcpC 1–507), expressing different N-terminal parts of TcpC. We also realized that the different constructs varied in their cellular distribution. Microscopy of the CFT073 transformants with higher resolution demonstrated that TcpC 1–150 was mainly located at the cell wall. This finding confirmed the predicted transmembrane domain in this part of the molecule (Figure 3A,B). The culture of CFT073 p(tcpC 1–150):eYFP:AMP in LB medium apparently increased the cell wall localization of TcpC 1–150 (Figure 3B). It appeared that TcpC 127–507 was mainly localized at the cell poles (Figure 3C) while TcpC 1–507 and TcpC 508–924 were homogeneously expressed (Figure 3D,E). Finally, TcpC 127–924 and full-length TcpC 1–924 again showed the polarized expression pattern observed with TcpC 127–507 (Figure 3F,G). Filamented bacteria could no longer be observed 24 h post induction (Figures S2 and S3). The principal expression pattern of the TcpC constructs within CFT073 observed at four hours post induction remained at 24 h post induction (Figure 4) with the exception of TcpC 127–507, which lost its pure polarized expression pattern, and TcpC 508–924, which now was expressed in specks (Figure 4D). In summary, overexpression of TcpC caused growth retardation and presumably impairment of cell division as indicated by filamented bacteria.

### 2.2. Endogenous Promoters Controlling the tcpC Gene

**Figure 5:**
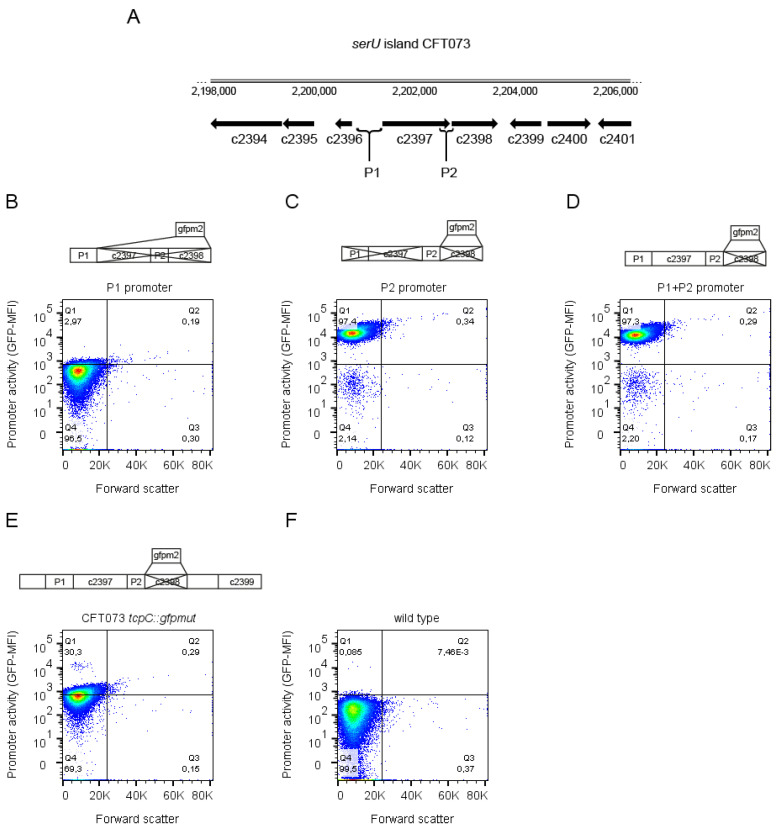
Genomic location and activity of putative promoter regions controlling the *tcpC* gene. The scheme depicts the central part of the *serU* island of CFT073. Top line illustrates the position of this part of the genome within the chromosome of CFT073. Black arrows illustrate gene sizes and the direction of gene transcription. The positions of the putative promotors 1 (P1) and 2 (P2) are also indicated (**A**). CFT073 was transformed either with the plasmids pPc2397:gfpmut2:KAN, pPc2398:gfpmut2:KAN or p(Pc2397–Pc2398):gfpmut2:KAN containing the promoter P1, or P2 or P1 plus P2, respectively, as indicated (**B**–**D**). In addition, we analyzed the chromosomal reporter strain CFT073 *tcpC::gfpmut2* (**E**). Untransformed, wild-type CFT073 served as negative control (**F**). Bacteria were incubated for four hours in M9-minimal medium containing glucose (0.4% = 22.2 mmol/L) and analyzed by flow cytometry directly after the incubation period without fixation. In addition to the depicted experiment, we repeated the experiment twice with identical results.

Based on our observation that TcpC exerts fundamental changes on CFT073, we concluded that the bacterium tightly controls the expression of TcpC. We, therefore, explored endogenous promoters putatively involved in the regulation of *tcpC* expression. We assumed that an untranslated region 5′ of the gene c2397, encoding for a hypothetical protein, might function as the promoter since this gene may form an operon with the *tcpC* gene (c2398) (Figure 5A). In addition, we also tested an untranslated region 5′ of the *tcpC* gene (c2398) as a putative promoter. We thus cloned a 645 bp DNA fragment starting at position 2200564 and ending at position 2201209 and a 240 bp element starting at position 2202535 and ending at position 2202775 of the chromosome of CFT073, respectively. We designated the 645 bp element as promoter 1 (P1) and the 240 bp element as promoter 2 (P2) (Figure 5A). We fused both putative promoter regions with the green fluorescent protein mut2 (*gfpmut2*) to generate plasmid-based reporter constructs. As shown in Figure 5B–D, we transformed CFT073 with three different plasmids containing either P1 or P2 or P1 plus P2 in each case fused to *gfpmut2*. In addition, we also replaced *tcpC* by *gfpmut2* on the chromosome of CFT073 generating the *tcpC*-deficient strain CFT073 *tcpC::gfpmut2* (Figure 5E). We cultured the four different CFT073 strains in glucose-containing M9-minimal medium and analyzed the expression of the reporter constructs and the size of the bacteria by flow cytometry. The activity of P1 was hardly detectable in comparison to CFT073 lacking a reporter construct (Figure 5B,F). In contrast, P2 was active and the presence of P1 did not diminish the activity of P2 (Figure 5C,D). The chromosomal reporter construct showed a clearly detectable activity although weaker than P2 as expected (Figure 5E). Glucose-containing M9-minimal medium did not influence the forward scatter behavior of the bacteria (Figure 5), indicating that the filamentation seen with induced expression results from overexpression of the TIR domain of TcpC.

**Figure 6:**
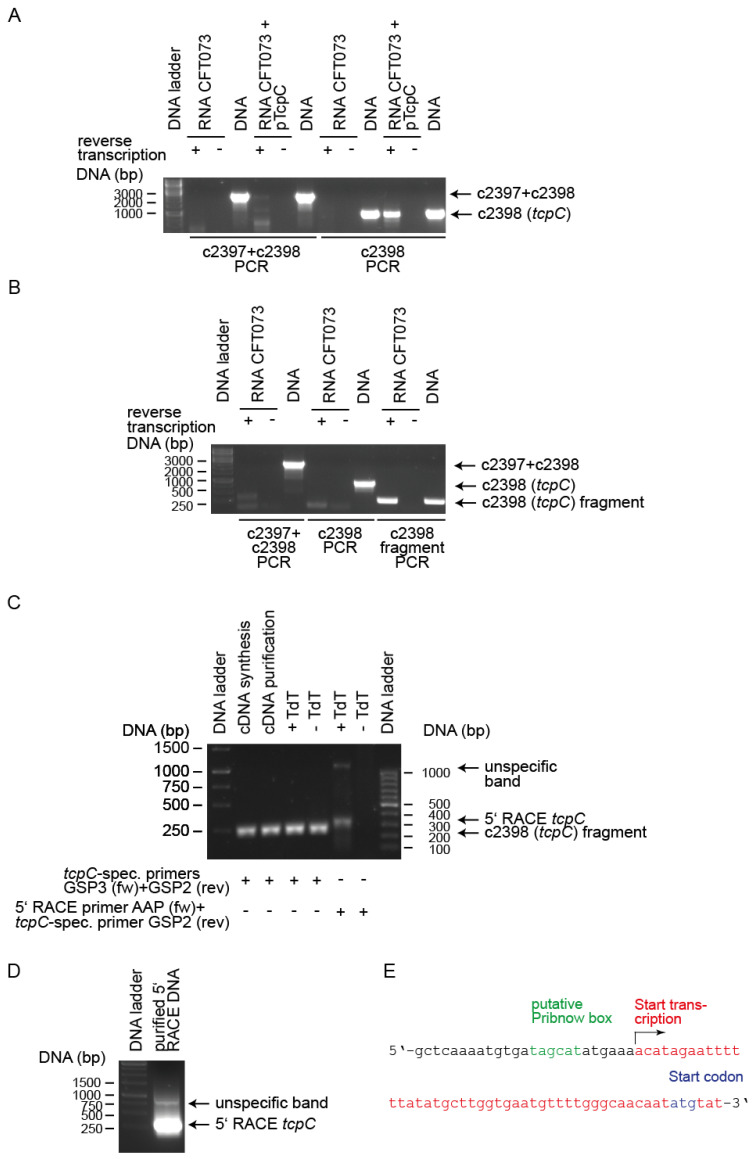
Detection of *tcpC* transcripts and genomic position of the P2 promoter. We cultured CFT073 or CFT073 transformed with the plasmid pTcpC overnight in M9-minimal medium containing glucose (0.4% = 22.2 mmol/L), pH8 until an OD_600_ of 0.65 was reached. (**A**) We prepared RNA and reverse transcribed or not (as indicated) using the primer c2398rev and performed a PCR using the primers c2398fw and c2398rev to amplify the almost complete *tcpC* (c2398) mRNA. We also used primers c2397fw and c2398rev to detect a long transcript encompassing the genes c2397 and c2398. (**B**) We cultured CFT073, prepared RNA and reverse transcribed or not as indicated and described in (**A**). PCR reactions were also performed as described in (**A**) but in addition we used the primer GSP3 (forward primer) and GSP2 (reverse primer) to amplify a shorter fragment of c2398. Genomic DNA of CFT073 served as the positive PCR control in (**A**,**B**). (**C**) For 5′ RACE PCR we synthesized cDNA using the primer GSP1. We performed a control PCR using the primers GSP3 (forward primer) and GSP2 (reverse primer) to detect *tcpC* (c2398) mRNA. To detect the start of the c2398 transcript we used the forward primer AAP and the reverse primer GSP2. We only detected a transcript post addition of oligo C by terminal deoxynucleotidyl transferase (TdT) as expected. (**D**) We re-amplified the 5′ RACE *tcpC* band depicted in (**C**) using the forward primer AUAP and GSP2 (rev. primer). (**E**) Sequencing of the PCR product depicted in (**D**) revealed that the transcript starts at position 2202778 of the CFT073 genome, or 44 bp 5′ of a possible start codon of c2398. Binding sites of all primers used for reverse transcription PCR and 5′ RACE PCR are depicted in Figure 12.

To provide further evidence for the existence of the P1 and P2 promoters, we analyzed RNA transcripts encompassing either c2397 and c2398 or 2398 alone. We used CFT073 or CFT073 transformed with the plasmid pTcpC containing a DNA fragment starting at 535 bp 5′ of the start codon of c2397 and ending at the stop codon of c2398. We used the latter strain to increase the sensitivity of the reverse transcription PCR. The strains were cultured in glucose-containing M9-minimal medium, pH8, which stimulates promoter activity (see below). While the long transcript was not detectable, we could demonstrate the transcript of the c2398 gene in case of the plasmid strain CFT073 pTcpC (Figure 6A). We used a new set of primers to amplify a shorter fragment of c2398 RNA post reverse transcription and could demonstrate a c2398 transcript in CFT073 (Figure 6B). We then performed a 5′RACE PCR to determine the transcription start of the 2398 transcript. We detected a terminal deoxynucleotidyl transferase (TdT)-dependent transcript (Figure 6C, compare lanes 5 and 6), which we could re-amplify with a second forward but the same reverse primer (Figure 6D). We sequenced this PCR product and found that transcription of c2398 starts 44 bp 5′ of a possible start codon of the c2398 RNA (Figure 6E). Interestingly, the c2398 RNA demonstrated in Figure 6A was obtained with the forward primer c2398fw binding 5′ of the transcription start demonstrated in Figure 6E with 16 of 22 bases. This may be compatible with the existence of a second promoter 5′ of the c2397 gene, although we could not demonstrate a long transcript presumably due to sensitivity issues.

**Figure 7:**
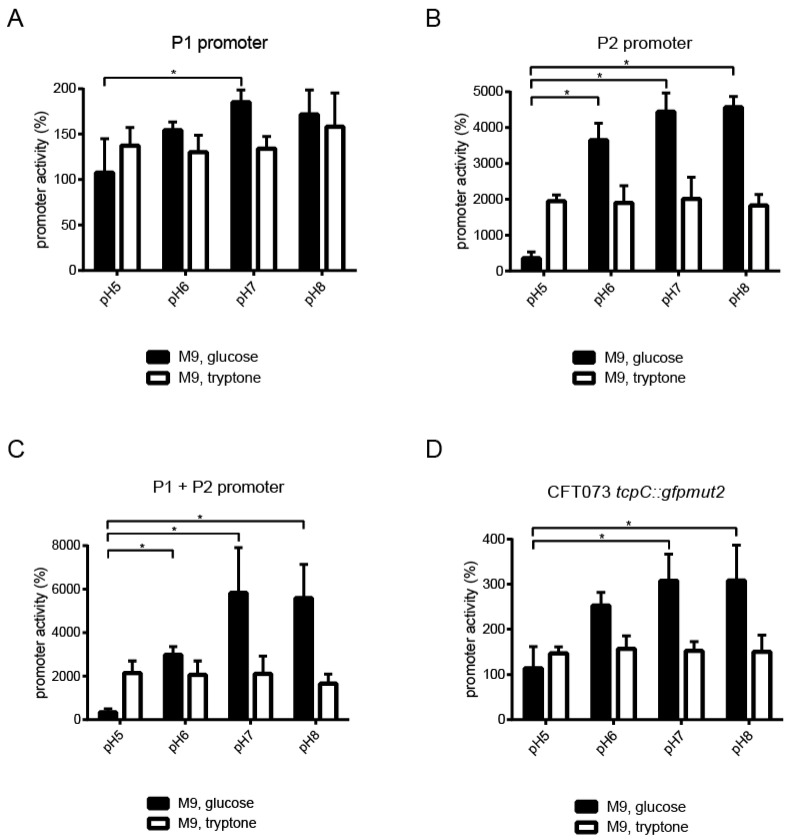
High pH induces the *tcpC* promoter. CFT073 was transformed either with the plasmids pPc2397:gfpmut2:KAN, pPc2398:gfpmut2:KAN or p(Pc2397–Pc2398):gfpmut2:KAN containing the promoter P1, or P2 or P1 plus P2, respectively (**A**–**C**). In addition, we analyzed the chromosomal reporter strain CFT073 *tcpC::gfpmut2* (**D**). We incubated bacteria overnight in M9-minimal medium-containing either glucose or tryptone at increasing pH as indicated and measured the expression of GFPmut2 by flow cytometry determining the mean fluorescence intensity (MFI). The values indicated by the bars represent three independent experiments and were normalized to MFI of wild-type CFT073 cultured in M9-minimal medium-containing glucose or tryptone at the pH tested. * *p* < 0.05, ANOVA post-hoc Tukey’s test.

We then explored which factors might induce *tcpC* promoters and analyzed the four different CFT073 strains in glucose- or tryptone-containing M9-minimal medium at different pH, since pH values may vary from 5.2 to 7.4 in the urinary tract. The experiment revealed that high pH strongly induced P2 but only weakly P1 if the strains were incubated in glucose- but not in tryptone-containing M9-minimal media (Figure 7A,B, Figure S4). P1 and P2 combined induced the reporter as strongly as P2 alone, demonstrating that P1 did not attenuate P2 (Figure 7C). High pH also induced the expression of *gfpmut2* in CFT073 *tcpC::gfpmut2*, again if cultured in glucose-containing M9-minimal medium (Figure 7D, Figure S4).

### 2.3. Glucose Induces the tcpC Promoter

**Figure 8:**
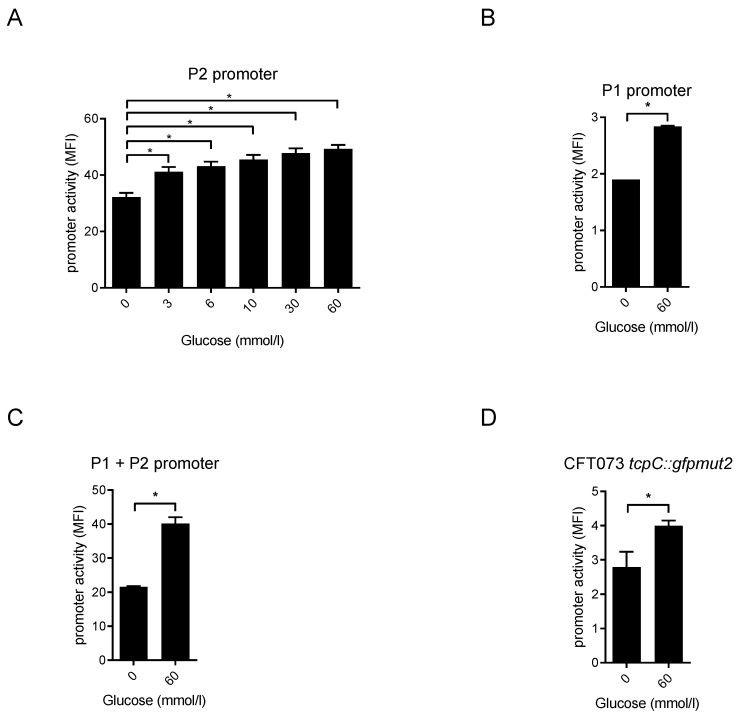
Glucose stimulates activity of the *tcpC* promoter. We cultured CFT073 carrying the plasmids described in Figure 5 in M9-minimal medium in the absence of glucose for 2 h, then added glucose in concentrations as indicated in the graphs for 3 h and measured the expression of GFPmut2 by flow cytometry. Since P2 was considerably more active than P1 we analyzed the reactivity of this promoter towards titrated amounts of glucose as indicated (**A**) and tested P1 (**B**) and P1 + P2 (**C**) only in the presence of the maximal glucose concentration. (**D**) demonstrates the chromosomal reporter strain CFT073 *tcpC::gfpmut2*. * *p* < 0.05, ANOVA post-hoc Tukey’s test. The values indicated by the bars represent three independent experiments.

Given the fact that increasing pH induced P1 and P2 only in the glucose-containing medium, we wondered whether glucose itself would induce *tcpC* promoters. Since P2 was more sensitive to pH changes, we tested this promoter first for its sensitivity against a range of glucose concentrations. A glucose concentration of 3 mmol/L induced P2 significantly and higher glucose doses increased its activity further in a dose-dependent manner (Figure 8A). Again, P1 reacted much weaker (Figure 8B) and the activity of P1 plus P2 was as strong as P2 alone (Figure 8C). The chromosomal reporter strain CFT073 *tcpC::gfpmut2* also increased the expression of its reporter glucose dependently and significantly (Figure 8D).

### 2.4. FeSO_4_ Dampens tcpC Promoter Activity

**Figure 9:**
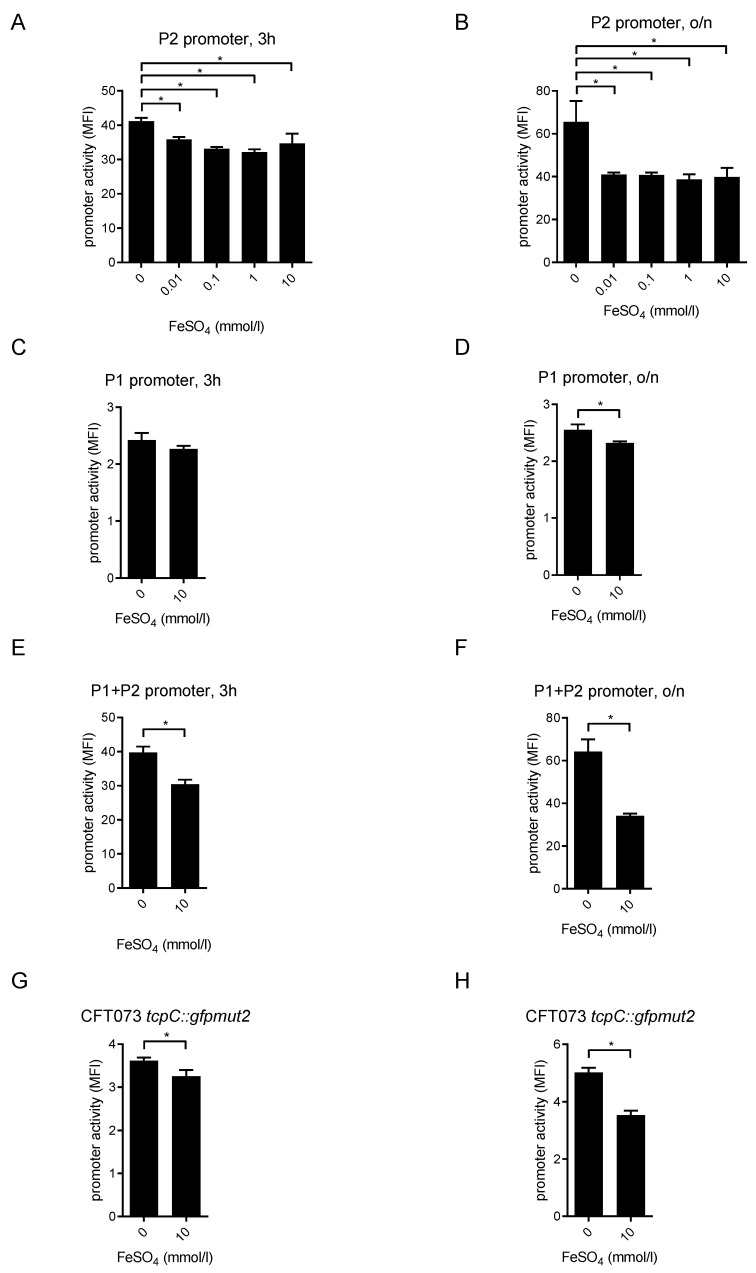
FeSO_4_ impairs the *tcpC* promoter. CFT073 transformed with the plasmids pPc2398:gfpmut2:KAN (**A**,**B**), or pPc2397:gfpmut2:KAN (**C**,**D**), or p(Pc2397–Pc2398):gfpmut2:KAN (**E**,**F**), or the mutant strain CFT073 *tcpC::gfpmut2* (**G**,**H**) were cultured in M9-minimal medium containing glucose in the presence of FeSO_4_ as indicated in the graphs. Fluorescence intensity of the reporter constructs was measured by flow cytometry 3 h (**A**,**C**,**E**,**G**) or overnight (**B**,**D**,**F**,**H**) post addition of FeSO_4_. * *p* < 0.05, ANOVA post-hoc Tukey’s test. In addition to the depicted experiment, we repeated the experiment twice with identical results.

**Figure 10:**
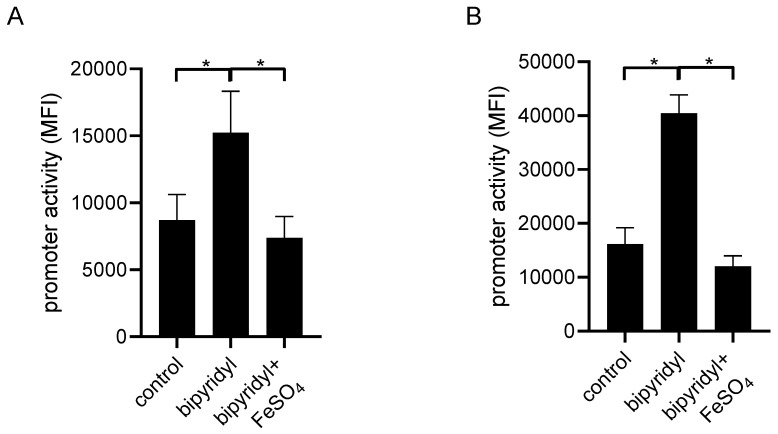
The iron chelator 2,2’-bipyridine induces the P2 promoter. CFT073 transformed with the plasmid pPc2398:gfpmut2:KAN were cultured in M9-minimal medium containing glucose in the presence of 2,2’-bipyridine (0.2 mmol/L) or 2,2’-bipyridine (0.2 mmol/L) plus FeSO_4_ (0.05 mmol/L), as indicated in the graphs. Fluorescence intensity of the reporter construct was measured by flow cytometry after 4 (**A**) or 24 h (**B**) of culture. The graphs depict three independent experiments. * *p* < 0.05, ANOVA post-hoc Tukey’s test.

Iron is essential for many bacterial pathogens, including UPECs, to maintain their own growth. To secure iron supply, UPECs including CFT073 possess siderophores, which bind Fe^3+^ and the generated complexes are subsequently transferred across the bacterial cell wall [20]. Since iron levels in urine are low [21], we wondered whether reduced iron levels would facilitate the expression of the *tcpC* gene. The expression of virulence factors such as TcpC might be an advantageous adaptation under these growth-limiting conditions. Incubation of the P2-reporter CFT073 strain with increasing FeSO_4_ concentrations demonstrated that the activity of P2 was reduced already 3 h post addition (Figure 9A). This effect was even more pronounced after overnight incubation with FeSO_4_ (Figure 9B). The activity of P1 was only weakly reduced by FeSO_4_, and the differences were only significant after an overnight incubation with FeSO_4_ (Figure 9C,D). In contrast, the combined activity of P1 and P2 was already reduced after 3 h incubation with FeSO_4_ and this was more pronounced after incubation overnight (Figure 9E,F). FeSO_4_ also reduced the activity of the chromosomal reporter strain CFT073 *tcpC::gfpmut2* significantly after incubation of 3 h and overnight (Figure 9G,H). To further analyze the influence of FeSO_4_ on the P2 promoter, we used the iron chelator 2,2’-bipyridine, which is also known to deplete intracellular iron from *E. coli* [22]. We found that incubation of the P2-reporter strain with the iron chelator significantly increased the activity of the P2 promoter (Figure 10). Co-administration of FeSO_4_ neutralized this effect (Figure 10). Taken together, FeSO_4_ clearly dampened the activity of the *tcpC* promoter and influenced plasmid or chromosomal reporter constructs similarly. Since increasing pH and glucose concentrations stimulated while FeSO_4_ impaired plasmid and chromosomal reporter constructs in a similar manner, we conclude that the regulation of the *tcpC* gene on the chromosome of CFT073 follows the rules observed with the plasmid reporter constructs.

### 2.5. Human Urine Induces the tcpC Promoter

**Figure 11:**
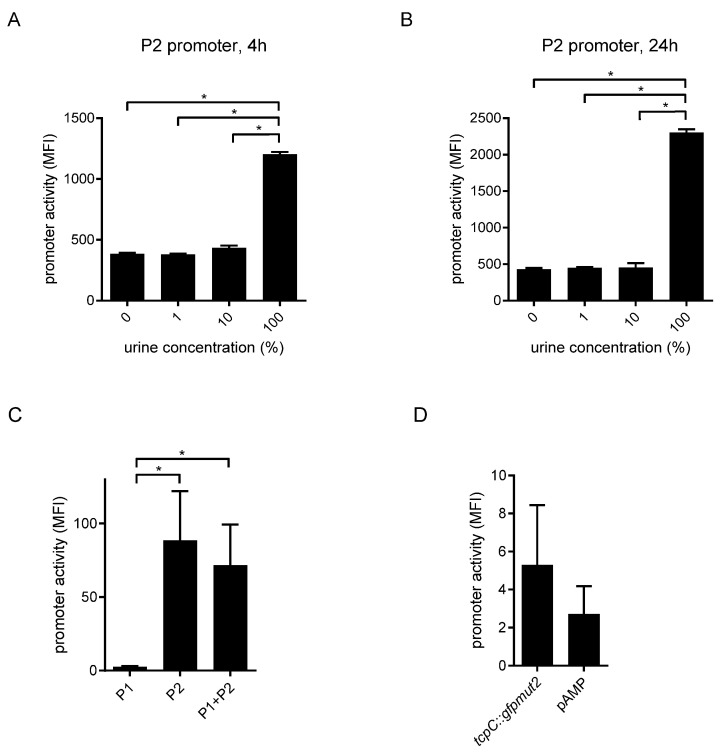
*TcpC* promoter is active in human urine. CFT073 pPc2398:gfpmut2:KAN was cultured in human urine diluted with M9-minimal medium supplemented with glucose as indicated in the graphs (**A**,**B**). Fluorescence intensity of the reporter constructs was measured by flow cytometry after 4 h (**A**) or 24 h of culture (**B**). CFT073 p(Pc2397:gfpmut2:KAN), CFT073 pPc2398:gfpmut2:KAN or CFT073 p(Pc2397–Pc2398):gfpmut2:KAN (**C**), or the mutant strain CFT073 *tcpC::gfpmut2* (**D**) were cultured overnight in urine (100%) and reporter activity was determined by flow cytometry. CFT073 pAMP served as negative control. * *p* < 0.05, ANOVA post-hoc Tukey’s test. Graphs in (**C**,**D**) depict the results of three independent experiments.

The bacterial burden of CFT073 in the urine and kidneys of infected mice was by several orders of magnitude higher compared to its *tcpC*-deficient mutant CFT073 *tcpC::KAN*, indicating that the *tcpC* promoter is active in urine during an infection [1,23]. To verify this assumption, we incubated our collection of reporter strains in urine of healthy human donors. Only pure urine induced the promoter P2 after an incubation period of 4 h and 24 h (Figure 11A,B). P1 was almost not induced while the combination P1 and P2 was activated as strongly as P2 alone (Figure 11C). Urine induced the chromosomal reporter strain CFT073 *tcpC::gfpmut2* to some extent, however the difference to the negative control strain CFT073 pAMP was not significant (Figure 11D). Due to the better signal to noise ratio of the plasmid reporter constructs and thus their higher sensitivity, we think that the latter constructs clearly demonstrate that P2 senses urine and induces the expression of the *tcpC* gene in urine.

## 3. Discussion

Our results demonstrate that overexpression of TcpC influences the UPEC strain CFT073 fundamentally in that growth is impaired and filamentation is induced. Thus, aside from TcpC’s influence on the innate immune system, the protein also provokes drastic changes on CFT073. Therefore, we explored the regulation of the *tcpC* gene. We found that a 240 bp region 5′ of the *tcpC* gene (c2398) served as the crucial promoter element, which we designated P2, while a second, functionally minor regulatory region, designated P1, is present on a 645 bp region 5′ of the gene c2397. We could map the site of transcription start of the c2398 mRNA to position 2202778 of the CFT073 genome, or 44 bp 5′ of a possible start codon. P2, and to a lower extent, P1, were induced by high pH, glucose concentrations and urine, while increasing concentrations of FeSO_4_ lowered promoter activity.

We also used the promoter prediction tool iPro70-FMWin, which predicts σ^70^ promoters with comparably high accuracy [24]. We focused on σ^70^ promoters since these are the most common promoters in *E. coli* [25]. IPro70-FMWin predicts that P1 and P2 contain a σ^70^ promoter with a probability score of 0.98 and 0.52, respectively. Both putative σ^70^ promoters are at a distance of around 480 and 150 bp to the translation start codon of c2397 and c2398, respectively. Most 5′ untranslated regions (UTR) in *E. coli* vary between 20 to 40 nucleotides in length, while some have longer 5′ UTRs between 100 and 290 nucleotides [25]. Thus, the length of 5′ UTR of the predicted σ^70^ promoter in P1 is unusual and our own results demonstrate a 5′UTR of only 44 bp in case of the P2 promoter.

The TIR domains of bacterial TIR containing proteins including TcpC and TcpB were recently shown to act as NAD^+^ hydrolases [19,26]. Overexpression of the TIR domain of TcpC reduced the intracellular bacterial concentration of NAD^+^ [19]. Since NAD^+^-dependent DNA ligases are present in all bacteria and are essential for growth, overexpression of TcpC may stop replication of CFT073 by a lack of NAD^+^-dependent DNA ligases [27]. However, our findings using mutant E244A TcpC do not support this conclusion since the mutant impaired the growth of CFT073-like wild-type TcpC.

So far, only overexpression of the TIR domain of TcpC induced bacterial filamentation, while *tcpC* promoter induction with glucose or high pH did not influence bacterial morphology. Filamentation of UPECs such as the UTI89 strain was described as a bacterial defense mechanism during urinary tract infection in mice and is presumably relevant for the entry and exit of bladder epithelial cells [28,29]. Interestingly, filamentation was not induced in mice lacking TLR4, indicating that innate immune responses trigger this morphologic change of the invading bacterium [28,30]. Functionally, filamented bacteria evaded phagocytosis by polymorphonuclear neutrophils [30]. Filamentation of UPECs was also observed in vitro upon infection of human bladder epithelial cells and in the urine of females with urinary tract infections, indicating that this bacterial escape mechanism is also operative in infected humans [31,32]. In the UTI89 strain the bacterial cell wall inhibitor SulA is active during filamentation and a *sulA*-deficient UTI89 mutant was attenuated to sustain a bladder infection in wild-type mice [28]. It remains to be seen whether other conditions such as an infection of host cells increases *tcpC* promoter activity and induces filamentation. Obviously, there are *tcpC*-independent pathways to induce filamentation since UTI89 does not contain a *tcpC* homolog; nevertheless, it is capable of adopting this morphologic change [30,33].

P2 was clearly more relevant than P1 for the induction of *tcpC* expression at all conditions tested. Thus, P2 was more sensitive to pH changes, increasing glucose or decreasing FeSO_4_ concentrations. It was also more active than P1 in human urine. Although P1 was only weakly induced, the changes were nevertheless significant. P1 never impaired the activity of P2 but instead the combination of both promoters was in almost all cases, except in the presence of urine, as active as the individual ones. Unfortunately, we could not demonstrate a polycistronic transcript encoding the mRNA of c2397 and c2398, but we also cannot rule out this possibility. In any case, we could not verify the relevance of P1 for the transcription of the *tcpC* (c2398) gene and we think that the mapped P2 promoter region is mainly responsible for *tcpC* transcription.

The fact that P2 was active in human urine is important since it demonstrates that the virulence factor TcpC may be expressed during a urinary tract infection of humans. This assumption is supported by the observation that the frequency of UPECs harboring the *tcpC* gene was particularly high in patients with pyelonephritis [1,16]. Thus, *tcpC* expression might be relevant in particular during an upper urinary tract infection. Moreover, CFT073 in comparison to its *tcpC*-deficient mutant multiplied to a significantly higher bacterial burden in murine urine and kidneys and only the CFT073 wild-type strain damaged kidneys during an urinary tract infection of mice [1]. However, other groups failed to detect expression of *tcpC* in *E. coli* strains isolated from the urinary tract of patients. Accordingly, analysis of eight different *E. coli* isolates by CFT073-specific microarrays and another five *E. coli* isolates by RNA-seq from patients with bacteriuria or uncomplicated urinary tract infection, respectively, revealed the induction of iron acquisition and peptide transport systems but not of *tcpC* [34,35]. However, in patients with cystitis the frequency of isolates harboring the *tcpC* gene is only around 21% [1] and due to the limited number of isolates analyzed the collection did not include a *tcpC*^+^ isolate.

Our results also indicate which urine properties might influence the expression of TcpC. On the one hand, urine contains only low amounts of iron and therefore the negative influence exerted by FeSO_4_ is not present in the urinary tract [21]. On the other hand, the pH of urine is around six, which supports the expression of TcpC. The pH of the kidneys equals seven and therefore expression of TcpC may be further facilitated. However, glucose levels of urine are below 1 mmol/l and our results demonstrate that high pH values only induce the expression of *tcpC* in the presence of glucose. Therefore, we assume that additional urine components influence the expression of *tcpC* gene during an infection.

UPEC strains like CFT073 are not only known to cause pyelonephritis—and the strain was originally isolated from a patient suffering from this infection—[36] but also urosepsis, the most devastating complication of a urinary tract infection. Blood, in contrast to urine, contains glucose levels required for *tcpC* expression and a pH of seven, which is also compatible with *tcpC* expression. Thus, we speculate that TcpC is also produced during urosepsis.

We published earlier that TcpC may contain a transmembrane domain in its N-terminal as predicted in silico [1]. We now confirm the prediction, as the truncated construct *tcpC* (1–150) was expressed at the bacterial cell wall. We also reported earlier that TcpC was secreted and this process was inhibited by the efflux pump inhibitor phenylalanine-arginine-β-naphtylamide (PAβN) [1]. Possibly, this N-terminal part of TcpC is crucial for its secretion. It is at present unclear why this expression pattern is not seen with the full-length construct. However, since TcpC is rapidly cleaved at least after transfection in eukaryotic cells and since the eYFP label is added at the C-terminus of TcpC, we may fail to observe this because the fluorescent label may be cleaved off inside CFT073 [13].

In summary, we demonstrate here that overexpression of *tcpC* impaired growth of the UPEC strain CFT073 and provoked its filamentation. We defined the location of the *tcpC* promoter 5′ of *tcpC* and c2397 and we explored its regulation: pH, glucose and urine switched the promoter on while FeSO_4_ switched it off.

## 4. Materials and Methods

### 4.1. Bacterial Strains

The UPEC strain CFT073 was purchased from ATCC (Manassas, VA, USA) [36]. The *tcpC*-deficient reporter strain CFT073 *tcpC::gfpmut2* was constructed using the λ-red system [37]. Briefly, we amplified the 5′UTR of *tcpC* fused to *gfpmut2* from pPc2398:gfpmut2:KAN containing this region and using primers SN10 (fw, 5′-TACTATCTCGAGGCAGGAGTCTATGGTAACG-3′) and SN19 (rev, 5′-GAAGCAGCTCCAGCCTACACTTATTTGTACAATTCATCC-3′). The latter contains the P1 site of pKD13, encoding the kanamycin resistance gene. The downstream region of *tcpC* was amplified from the CFT073 genome with primers SN22 (fw, 5′-ATAGTAGAGACCGGAAGACACGGATTCCATG-3′) having an overhang with the P4 site of pKD13 and SN23 (rev, 5′-TGCCCATTAACATCACCATC-3′). Subsequently, the kanamycin cassette was amplified from pKD13 using the above fragments and outer primers SN10 and SN23. The obtained donor DNA was transformed into recipient strain CFT073 expressing the λ-red system from helper plasmid pKD46 by electroporation. Expression of lambda red recombinase was induced by treatment with 100 mM arabinose at 30 °C. Transformants were recovered in S.O.C medium (Invitrogen Corp., Paisley, UK). After selection on kanamycin (50 mg/mL), the resistance cassette was flipped out using the curable temperature-sensitive plasmid pCP20 encoding FLP recombinase.

### 4.2. Plasmids

All plasmids generated for this study are listed in Table 1. We generated the plasmid pTcpC, which contains a DNA fragment starting 535 bp 5′ of the start codon of c2397 and ending at the stop codon of c2398. We cloned full-length *tcpC* into the non-leaky bacterial expression vector pASK-IBA5plus (IBA GmbH, Göttingen) containing a N-terminal Strep-tag and *tcpC:eYFP* into pASK-IBA3plus (IBA GmbH, Göttingen) containing a C-terminal Strep-tag. We also cloned full-length E244A-mutated *tcpC* into pASK-IBA5plus. We constructed three different gfpmut2-reporter constructs to define the endogenous *tcpC* promoter, five different *tcpC*-truncated constructs and one full-length *tcpC* construct, which were IPTG-inducible and transformed into CFT073. Sequences of primers used are listed in Table 2, the primer combinations to amplify P1, P2, P1 plus P2, full-length *tcpC* and truncated constructs of *tcpC* in Table 3. Restriction enzymes used to clone amplicons into the appropriate plasmid are also described in Table 2 and Table 3. In total, 100 μL of electro-competent CFT073 and 0.1–0.3 μL of a plasmid-miniprep were mixed and the plasmids electroporated (5 ms, 1700 V, Multiporator, Eppendorf, Germany) into CFT073. Subsequently, 100 μL LB medium was added, and the bacterial suspension was cultured on LB agar plates supplemented with kanamycin or ampicillin. We picked single colonies after overnight incubation. The correct composition of all plasmids was verified by sequencing (GATC, Konstanz, Germany).

### 4.3. Reagents and Culture Media

Isopropyl-β-D-thiogalactopyranosid (IPTG) came from Applichem GmbH (Darmstadt, Germany), anhydrotetracycline (ATc) from Toku-e (Gentaur GmbH, Aachen, Germany), Luria-Bertani broth (LB) and LB agar plates were purchased from Roth (Karlsruhe, Germany). Components for the M9-minimal medium (di-sodium-hydrogenphosphate (33.9 g/L), potassium-dihydrogenphosphate (15 g/L), sodium chloride (2.5 g/L), ammonium-chloride (5 g/L)) came from Merck (Darmstadt, Germany). This medium was either supplemented with glucose (0.4%), thiamin (10 µg/mL, Roth) and nicotic acid (0.0025%, Roth) or tryptone (0.2%, BD Chemicals, Greenwood Village, CO, USA). 2,2′-bipyridine, 5 g, was bought from Roth. Human urine was donated by healthy individuals. The urine was filtered (0.2 µm pore size) and used on the day of donation.

### 4.4. Culture of Bacteria

The *tcpC*-deficient reporter strain CFT073 *tcpC::gfpmut2* and plasmid-transformed CFT073 strains were cultured overnight in M9-minimal medium containing glucose or in case of the *tcpC* promoter stimulation with urine in LB medium in the presence of ampicillin (100 μg/mL) or kanamycin (25 μg/mL); wild-type CFT073 was cultured in the absence of antibiotics. After three wash steps, bacterial densities were adjusted to an OD_600_ of 0.5, diluted and further cultured in LB or M9-minimal medium supplemented with either glucose, thiamin and nicotic acid or tryptone as indicated in the figure legends.

### 4.5. Growth Determination

Bacterial growth was determined by measuring the optical density using an OD_600_ DiluPhotometer (IMPLEN, Munich, Germany).

### 4.6. Reporter Assays

CFT073 strains transformed with the different truncated or full-length *tcpC* reporter plasmids were induced with IPTG (20 μmol/L) and incubated for 4 or 24 h. At each time point, bacteria were fixed with PFA/PBS (1%, 30 min, room temperature). After several wash steps with PBS, bacterial pellets were immobilized on agarose pads (1%) and analyzed with a Leica Leitz DMR fluorescence microscope (Leica, Wetzlar, Germany) equipped with an Orca flash 4.0 LT camera (Hamamatsu Photonics Deutschland GmbH, Herrsching am Ammersee, Germany) using an excitation wavelength of 524 nm.

We used flow cytometry (FACScan, BD Biosciences, Germany) to explore the fluorescence activity of CFT073 strains transformed with the three different *tcpC*-promoter reporter plasmids and the *tcpC*-deficient CFT073 *tcpC::gfpmut2* strain. We analyzed the data with FlowJo (Ashland, OR, USA).

### 4.7. Reverse Transcription PCR and 5′ RACE PCR

To detect a polycistronic mRNA of the hypothetical gene c2397 and c2398 (*tcpC*) or just the mRNA of c2398, RNA was extracted from CFT073 and CFT073 p(TcpC). Bacterial cultures of CFT073 and CFT073 + p(TcpC) were grown overnight in glucose-M9-minimal medium pH8 to induce the promoter. The medium for CFT073 p(TcpC) contained chloramphenicol (34 μg/mL). At mid-logarithmic phase, around 2.5 mL of the culture (corresponding to approximately 1.5 × 10^9^ bacteria) were harvested, washed three times with Dulbecco’s Phosphate-Buffered Solution (DPBS) and resuspended in 100 µL DPBS plus 250 µL DNA/RNA Shield (Zymo Research, Freiburg, Germany). After incubating the samples for five minutes and centrifugation (10 min, 5000 g), the supernatant was removed and 200 µL TE-buffer (10 mM Tris·Cl, 1 mM EDTA, pH8) containing 1 mg/mL lysozyme was added. The samples were vortexed and incubated for five minutes under shaking and then mixed with 700 μL RLT-buffer (RNeasy Mini Kit (Qiagen, Hilden, Germany)) plus 7 µL β-mercaptoethanol. After vortexing and centrifuging (2 min, 16000 g) the samples, the supernatant was mixed with 500 µL ethanol (100%). The RNA was then extracted with the RNeasy Mini Kit (Qiagen). During the procedure, two on-column DNase digests were conducted (RNase-Free DNase Set (Qiagen)) and the purified RNA was eluted in 50 µL RNAse-free H_2_O. Then, another DNase digest was performed in solution according to the kit manual to get rid of residual DNA traces. The DNase was removed with a second column purification (RNeasy Mini Kit) and the pure RNA was eluted in 50 µL RNAse free H_2_O. RNA quality was examined with gel electrophoresis of 10 µL sample on a 1% agarose-TAE agarose gel and ethidium bromide staining (60 min, 7 V/cm).

We also took 20 µL aliquots of the overnight cultures, incubated them at 95 °C for 10 min and stored them at −20 °C as PCR positive controls.

**Figure 12:**
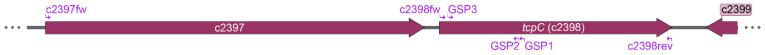
Binding sites of forward and reverse primers for reverse transcription PCR and 5′ RACE PCR.

The RNA was transcribed to cDNA with the RevertAid First Strand cDNA Synthesis Kit (Thermo Scientific, Karlsruhe, Germany) and the Primer c2398rev (Table 2, Figure 12) that binds at the end of c2398. For each of the two samples, two reactions with and without reverse transcriptase (+/−RT) were set up to test for DNA contamination in the PCR. GAPDH-RNA served as a positive control.

In total, 2 µL of the +/− RT-cDNA and boiled bacteria samples (positive control) were then amplified using two different primer sets spanning either the entire operon (c2397fw—c2398rev, Table 2, Figure 12) or just c2398 (c2398fw—c2398rev, Table 2, Figure 12). Then 10 µL of PCR product was analyzed on an 1% agarose gel and ethidium bromide staining (60 min, 7 V/cm).

The 5′ RACE PCR was performed using the kit “5′ RACE System for Rapid Amplification of cDNA Ends, Version 2.0” (Invitrogen, ThermoFisher, Waltham, MA, USA). In brief, the extracted RNA was reverse transcribed into cDNA with SuperScript II Reverse Transcriptase and the primer GSP1 (Table 2, Figure 12), binding 336 bp downstream of the putative *tcpC* start codon. The RNA was degraded with RNase Mix and the cDNA purified with a S.N.A.P column to remove dNTPs, GSP1 and proteins. In the next step, a poly-C-tail was added to the 5′-end of the cDNA with the enzyme terminal deoxynucleotidyl transferase (TdT). A negative control without TdT was included. The tailed product was then amplified in a PCR using the poly-G and deoxyinosine-containing primer AAP (Table 2) that binds to the poly-C tail and the nested primer GSP2 (Table 2, Figure 12) that binds the cDNA strand. Additionally, a second positive control PCR was performed using GSP3 (Table 3, Figure 12) that binds 34 bp downstream of the putative start codon and GSP2 (Figure 12). Aliquots of the PCR products were analyzed on an agarose gel and the rest of the RACE-PCR sample was purified with the QIAquick PCR Purification Kit (Qiagen). To generate enough specific product, the product was re-amplified in a second PCR with the AUAP primer (Table 2) which was homologous to the 5′-part of the AAP-primer and GSP2 (Figure 12). We purified the PCR product via gel electrophoresis and extracted the amplicon using the kit NucleoSpin Gel and PCR Clean-up (Machery-Nagel GmbH & Co. KG, Düren, Germany). The purified product was then analyzed by Sanger-sequencing (LGC Genomics GmbH, Germany) with the primer GSP2 (Figure 12).

### 4.8. Statistics

We used Prism 6.07 (GraphPad software, San Diego, CA, USA) to analyze experimental results for statistically significant differences. One-way ANOVA and Tukey’s test as post-hoc tests served to compare the experimental groups. We considered *p* values of less than 0.05 to be significant.

## Figures and Tables

**Table 1:** Plasmids generated and transformed into CFT073.

Bacterial Host	Construct	Plasmid Back Bone	Resist-Ance	Plasmid Name	Function
CFT073	5′UTR-c2397-c2398	pACYC-184	cm	pTcpC [1]	contains a DNA fragment starting at 535 bp 5′ of the start codon of c2397 and ends at the stop codon of c2398.
CFT073	*tcpC*-full-length (bp 1–924)-Strep-tag	pASK-IBA5plus	amp ^†^	p(Strep-tag tcpC 1-924):AMP	non-leaky, ATc-inducible expression of Strep tag-TcpC
CFT073	*tcpC*-full-length (bp 1–924) Strep-tag, E244A	pASK-IBA5plus	amp ^†^	p(Strep-tag E244A tcpC 1-924):AMP	non-leaky, ATc-inducible expression of Strep tag-E244A mutated TcpC
CFT073	*tcpC*-full-length(bp 1–924)-eYFP	pASK-IBA3plus	amp	p(tcpC 1-924):eYFP:Strep-tag:AMP	non-leaky, ATc-inducible expression of TcpC-YFP-Strep-tag
CFT073	Pc2397:gfpmut2	pUA66 [38]	kan ^‡^	pPc2397:gfpmut2:KAN	P1 reporter
CFT073	Pc2398:gfpmut2	pUA66	kan	pPc2398:gfpmut2:KAN	P2 reporter
CFT073	Pc2397–Pc2398:gfpmut2	pUA66	kan	p(Pc2397-Pc2398):gfpmut2:KAN	P1+P2 reporter
CFT073	-	pTrc99A [39]	amp	pAMP	negative control
CFT073	*tcpC*-fragment (bp 1–150)-eYFP	pDK112 [40]	amp	p(tcpC 1–150): eYFP:AMP	overexpression of YFP-labeled *tcpC* bp 1–150
CFT073	*tcpC*-fragment (bp 1–507)-eYFP	pDK112	amp	p(tcpC 1–507): eYFP:AMP	overexpression of YFP-labeled *tcpC* bp 1–507
CFT073	*tcpC*-fragment (bp 127–507)-eYFP	pDK112	amp	p(tcpC 127–507): eYFP:AMP	overexpression of YFP-labeled *tcpC* bp 127–507
CFT073	*tcpC*-fragment (bp 508–924)-eYFP	pDK112	amp	p(tcpC 508–924): eYFP:AMP	overexpression of YFP-labeled *tcpC* bp 508–924
CFT073	*tcpC*-fragment (bp 127–924)-eYFP	pDK112	amp	p(tcpC 127–924): eYFP:AMP	overexpression of YFP-labeled *tcpC* bp 127–924
CFT073	*tcpC*-full-length (bp 1–924)-eYFP	pDK112	amp	p(tcpC 1–924): eYFP:AMP	overexpression of YFP-labeled *tcpC* bp 1–924

^†^ ampicillin, ^‡^ kanamycin.

**Table 2:** Primer names and sequences used in this study. Primer sequences including restriction sites are indicated by lower case letters.

Designation	Sequence
pASK-IBA5 16_tcpC_BsaI_fw	5′-ATGGTAggtctcAGCGCCGTGATAGCATATGAAA-ACATAG-3′
pASK-IBA5 16_tcpC_BsaI_rev	5′-ATGGTAggtctcATATCATCTTCTCCTGTATGCT-ATTTC-3′
pASK-IBA3_tcpC_BsaI_fw	5′-ATGGTAggtctcAAATGGTGATAGCATATGAAAA-CATAG-3′
pASK-IBA3_eYFP_BsaI_rev	5′-ATGGTAggtctcAGCGCTCTTGTACAGCTCGTCC-ATGCCG-3′
c2397 5’UTR_XhoI_fw	5′-TACTATctcgagCACCTCTTGCTGTTTATACG-3′
c2397 5’UTR_BamHI_rev	5′-ATAGTAggatccGCCATTAAAAATATAATCTC-3′
c2398 5’UTR_XhoI_fw	5′-TACTATctcgagGCAGGAGTCTATGGTAACG-3′
c2398 5’UTR_BamHI_rev	5′-ATAGTAggatccCATATGCTATCACATTTTGAG-3′
tcpC bp 1_NcoI_fw	5′-TACTATccatgGTGATAGCATATGAAAACATAG-3′
tcpC bp 150_BamHI_rev	5′-ATAGTAggatccCTCTTTGGTTTTTAGGTGCTG-3′
tcpC bp 924_BamHI_rev	5′-ATAGTAggatccTCTTCTCCTGTATGCTATTTCA-G-3′
tcpC bp 508_NcoI_fw	5′-TACTATccatggACTATGATTTTTTCATATCC-3′
tcpC bp 507_BamHI_rev	5′-ATAGTAggatccCGTATTATTGTTATCTTGC-3′
tcpC bp 127_NcoI_fw	5′-TACTATccatggGGAAACAGCACCTAAAAACC-3′
c2397fw	5‘-ATGGCGATTTTTCATCTG-3‘
c2398fw	5‘-GTGATAGCATATGAAAACATAG-3‘
c2398rev	5‘-CTTCTCCTGTATGCTATTTC-3‘
GSP1	5‘-CTTTGCCTCAACCTCCTT-3‘
GSP2	5‘-CTCCCATCTATAATCGTGGAT-3‘
GSP3	5‘-GCTTGGTGAATGTTTTGG-3‘
AAP (Abridged Anchor Primer)	5‘-GGCCACGCGTCGACTAGTACGGGIIGGGIIGGGIIG-3‘
AUAP (Abridged Universal Amplification Primer)	5‘-GGCCACGCGTCGACTAGTAC-3‘

**Table 3:** Primer combinations used to amplify P1, P2, P1 plus P2, *tcpC* and different truncated *tcpC* constructs. Restriction enzymes were used as indicated in primer names to clone amplicons into the appropriate plasmids.

Construct	Forward Primer	Reverse Primer
Strep tag-*tcpC*-full-length (bp 1–924)	pASK-IBA5 16_tcpC_BsaI_fw	pASK-IBA5 16_tcpC_BsaI_rev
*tcpC*-full-length (bp 1–924)-eYFP	pASK-IBA3_tcpC_BsaI_fw	pASK-IBA3_eYFP_BsaI_rev
Pc2397:gfpmut2	c2397 5’UTR_XhoI_fw	c2397 5’UTR_BamHI_rev
Pc2398:gfpmut2	c2398 5’UTR_XhoI_fw	c2398 5’UTR_BamHI_rev
Pc2397–Pc2398:gfpmut2	c2397 5’UTR_XhoI_fw	c2398 5’UTR_BamHI_rev
*tcpC*-fragment (bp 1–150)-eYFP	tcpC bp 1_NcoI_fw	tcpC bp 150_BamHI_rev
*tcpC*-fragment (bp 1–507)-eYFP	tcpC bp 1_NcoI_fw	tcpC bp 507_BamHI_rev
*tcpC*-fragment (bp 127–507)-eYFP	tcpC bp 127_NcoI_fw	tcpC bp 507_BamHI_rev
*tcpC*-fragment (bp 508–924)-eYFP	tcpC bp 508_NcoI_fw	tcpC bp 924_BamHI_rev
*tcpC*-fragment (bp 127–924)-eYFP	tcpC bp 127_NcoI_fw	tcpC bp 924_BamHI_rev
*tcpC*-full-length (bp 1–924)-eYFP	tcpC bp 1_NcoI_fw	tcpC bp 924_BamHI_rev

## Data Availability

Data available on request from the authors.

## Supplementary Materials

The following are available online at https://www.mdpi.com/article/10.3390/pathogens10050549/s1, Figure S1. The TIR-domain of TcpC induces filamented bacteria. Microphotographs represent the corresponding light microscopy images of Figure 2. Figure S2. The filamentation of CFT07 induced by the TIR-domain of TcpC is no longer detectable 24 hours post induction.Bacteria were transformed as described in Figure 2 but analyzed 24 hours post induction with IPTG by fluorescence-microscopy. Note that CFT073 transformed with p(tcpC 508–924):eYFP:AMP (E) with p(tcpC 127–924):eYFP:AMP (F) and p(tcpC 1–924):eYFP:AMP (G), respectively, is no longer filamented. We repeated the experiment once with identical results. Figure S3. The filamentation of CFT07 induced by the TIR-domain of TcpC is no longer detectable 24 hours post induction. Microphotographs represent the corresponding light microscopy images of Figure S2. Figure S4. pH7 induces the promoter P1 and the chromosomal reporter construct significantly. We tested CFT073 transformed with the plasmids pPc2397:gfpmut2:KAN containing the promoter P1 (A), the chromosomal reporter strain CFT073 *tcpC::gfpmut2* (B) and CFT073 (C). Bacteria were incubated overnight in M9-minimal medium containing glucose at a pH of 7. We determined expression of GFPmut2 by flow cytometry and determined the mean fluorescence intensity (MFI). The values indicated by the bars represent three independent experiments (D). * *p* < 0.05, ANOVA posthoc Tukey.
