# Supplementary material for: Regulation of Expression of the TIR-Containing Protein C Gene of the Uropathogenic Escherichia coli Strain CFT073
Source: Pathogens. 2021 May 1;10(5):549. doi: 10.3390/pathogens10050549 (PMC8147327; doi:10.3390/pathogens10050549)
Supplement: Supplementary file 1 [file pathogens-10-00549-s001.zip › pathogens-1036169-supplementary.pdf]

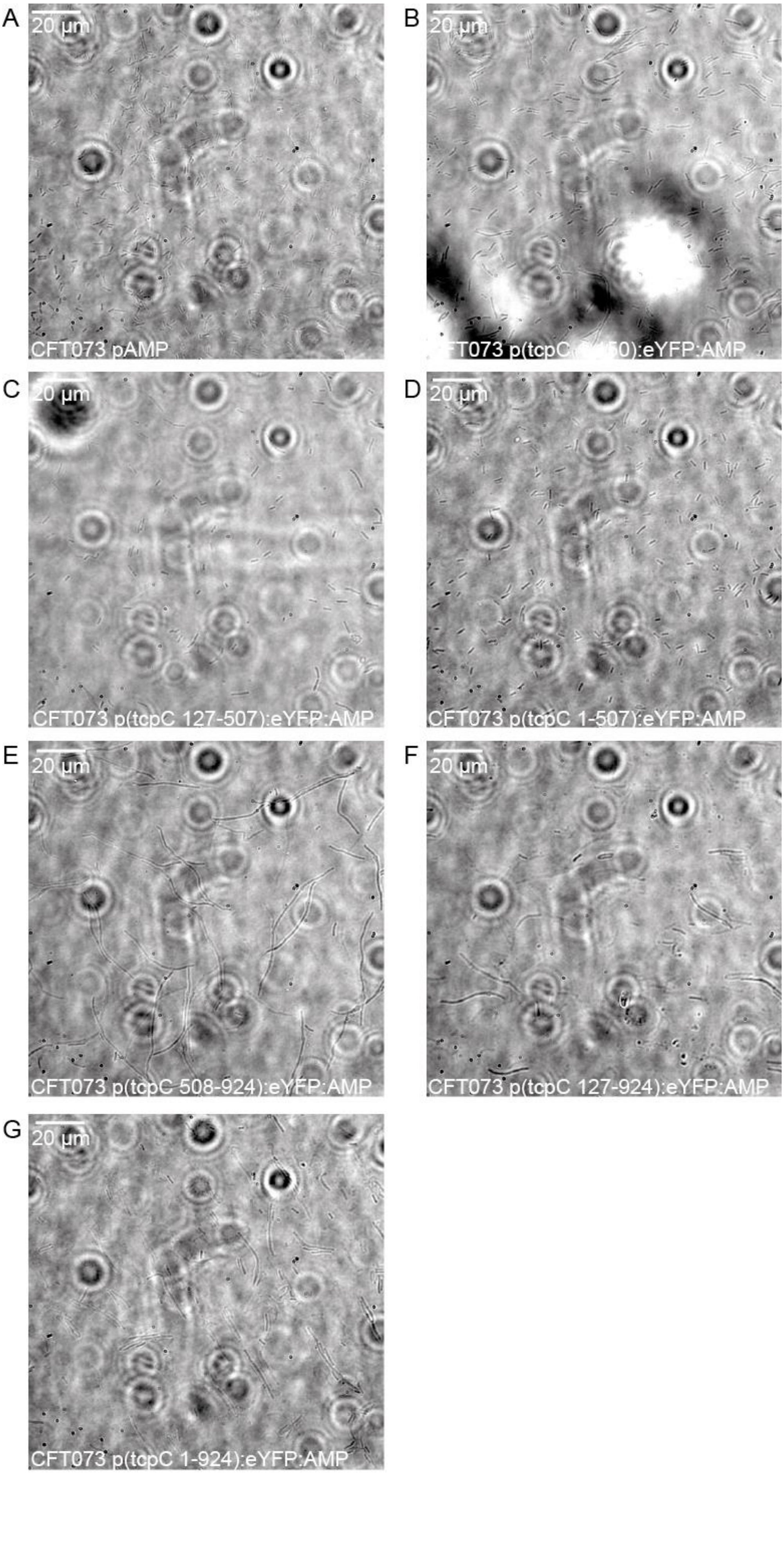
A
20 µm
CFT073 pAMP
B
20 µm
C
20 µm
CFT073 p(tcpC 127-507):eYFP:AMP
D
20 µm
CFT073 p(tcpC 1-507):eYFP:AMP
E
20 µm
CFT073 p(tcpC 508-924):eYFP:AMP
F
20 µm
CFT073 p(tcpC 127-924):eYFP:AMP
G
20 µm
CFT073 p(tcpC 1-924):eYFP:AMP

Figure S1. The TIR-domain of TcpC induces filamented bacteria. Microphotographs represent the corresponding light microscopy images of Fig. 2.

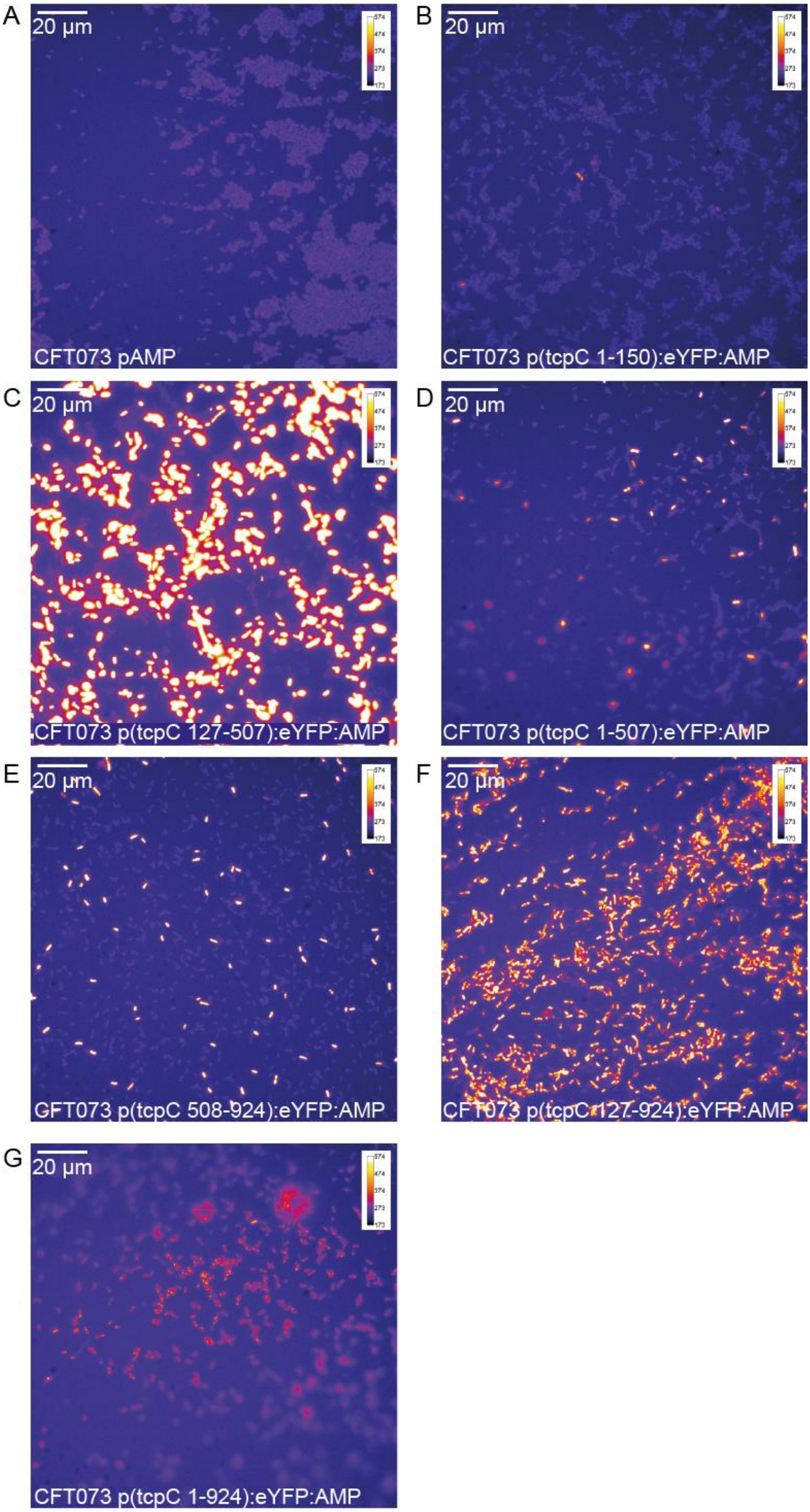
A
20 µm
CFT073 pAMP
B
20 µm
CFT073 p(tcpC 1-150):eYFP:AMP
C
20 µm
CFT073 p(tcpC 127-507):eYFP:AMP
D
20 µm
CFT073 p(tcpC 1-507):eYFP:AMP
E
20 µm
CFT073 p(tcpC 508-924):eYFP:AMP
F
20 µm
CFT073 p(tcpC 127-924):eYFP:AMP
G
20 µm
CFT073 p(tcpC 1-924):eYFP:AMP

Figure S2. The filamentation of CFT07 induced by the TIR-domain of TcpC is no longer detectable 24 hours post induction. Bacteria were transformed as described in Fig. 2 but analyzed 24 hours post induction with IPTG by fluorescence-microscopy. Note that CFT073 transformed with p(tcpC 508-924):eYFP:AMP (E) with p(tcpC 127-924):eYFP:AMP (F) and p(tcpC 1-924):eYFP:AMP (G), respectively, is no longer filamented. We repeated the experiment once with identical results.

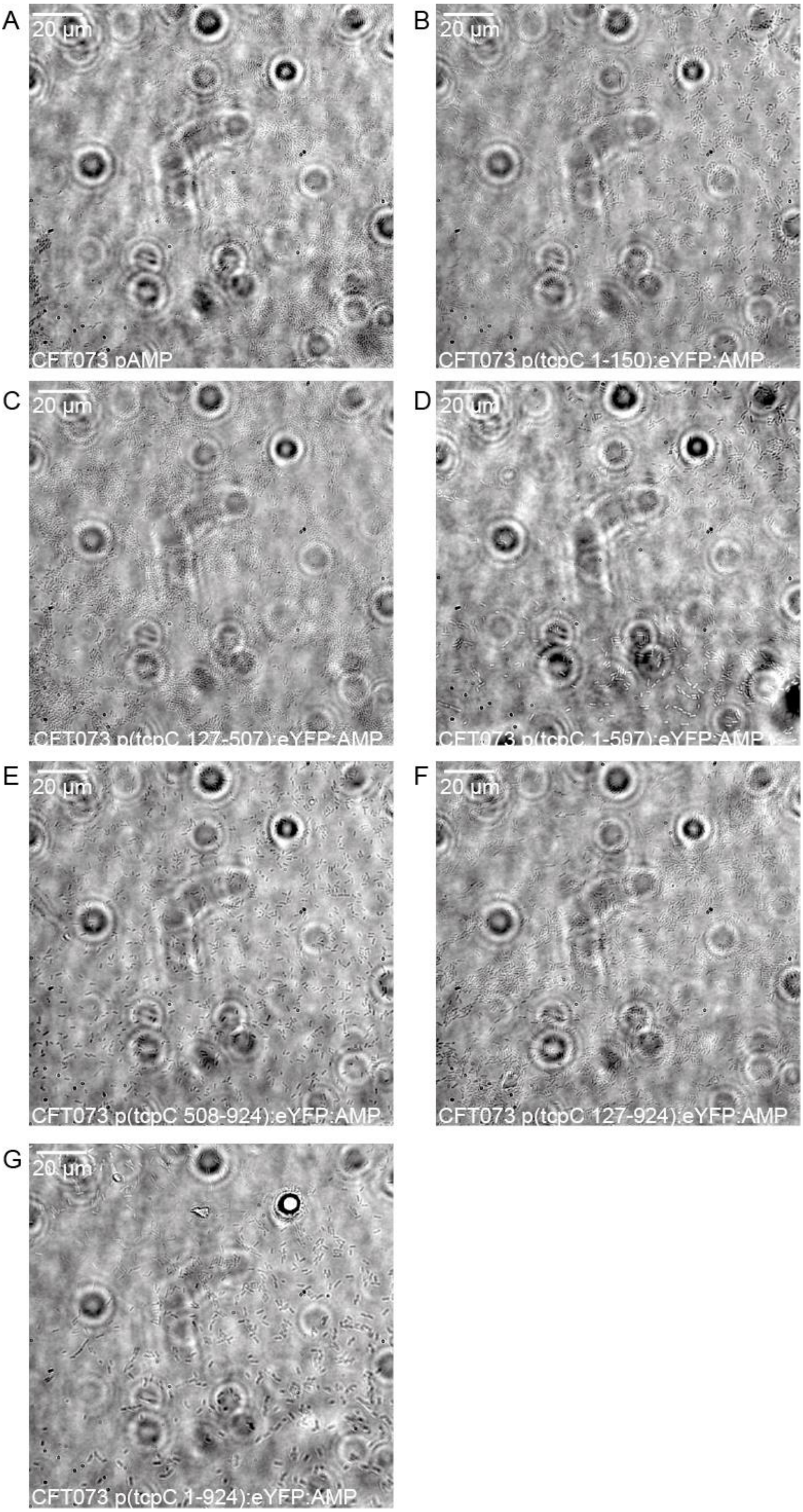
A
20 µm
CFT073 pAMP
B
20 µm
CFT073 p(tcpC 1-150):eYFP:AMP
C
20 µm
CFT073 p(tcpC 127-507):eYFP:AMP
D
20 µm
CFT073 p(tcpC 1-507):eYFP:AMP
E
20 µm
CFT073 p(tcpC 508-924):eYFP:AMP
F
20 µm
CFT073 p(tcpC 127-924):eYFP:AMP
G
20 µm
CFT073 p(tcpC 1-924):eYFP:AMP

Figure S3. The filamentation of CFT07 induced by the TIR-domain of TcpC is no longer detectable 24 hours post induction. Microphotographs represent the corresponding light microscopy images of Fig. S2.

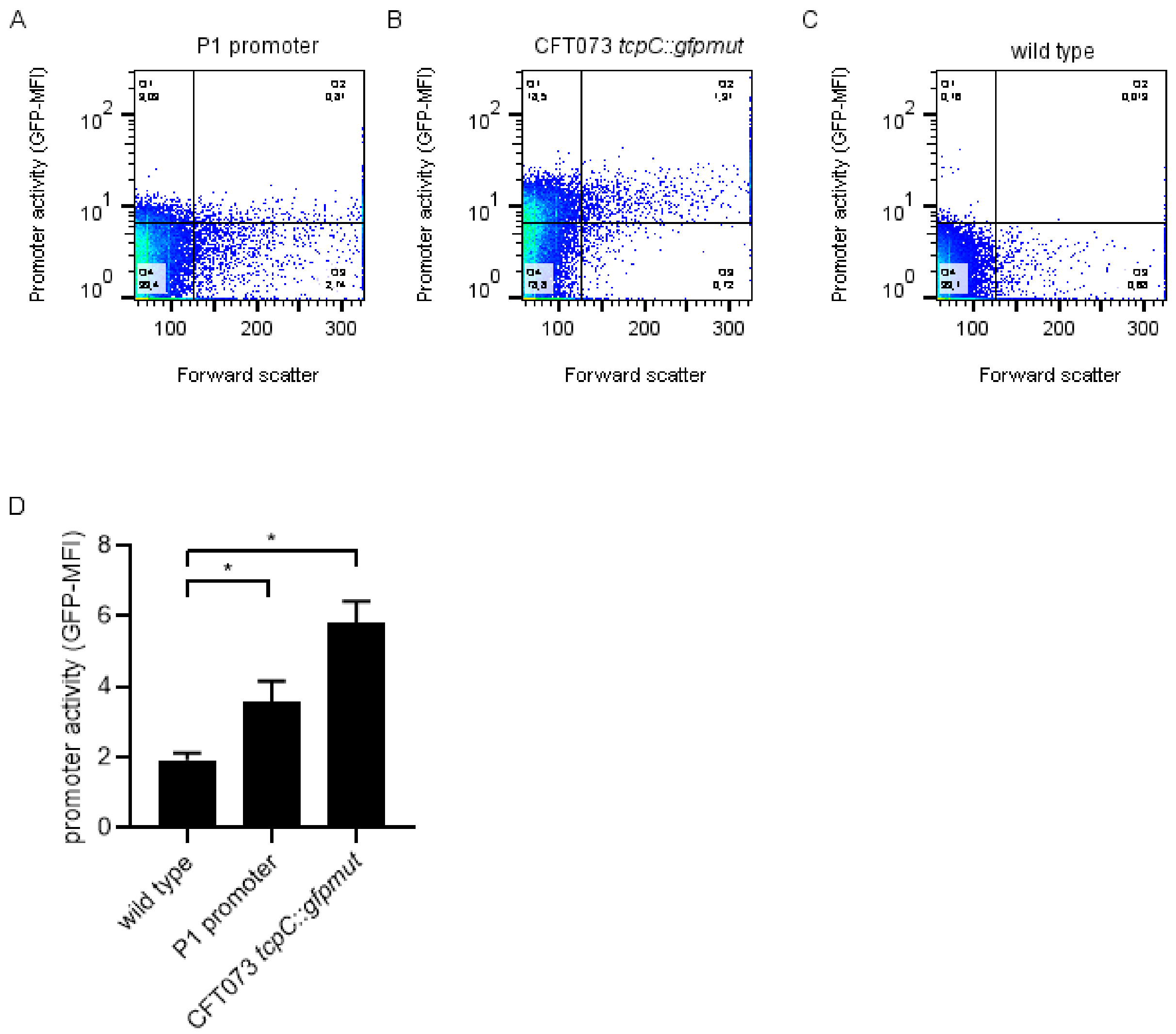

Figure S4. pH7 induces the promoter P1 and the chromosomal reporter construct significantly. We tested CFT073 transformed with the plasmids pPc2397:gfpmut2:KAN containing the promoter P1 (A), the chromosomal reporter strain CFT073 *tcpC::gfpmut2* (B) and CFT073 (C). Bacteria were incubated overnight in M9-minimal medium containing glucose at a pH of 7. We determined expression of GFPmut2 by flow cytometry and determined the mean fluorescence intensity (MFI). The values indicated by the bars represent three independent experiments (D). **P*<0.05, ANOVA posthoc Tukey.
